# Quality communication can improve patient-centred health outcomes among older patients: a rapid review

**DOI:** 10.1186/s12913-023-09869-8

**Published:** 2023-08-22

**Authors:** Samer H. Sharkiya

**Affiliations:** Faculty of Graduate Studies, Arab American University, 13 Zababdeh, P.O Box 240, Jenin, Palestine

**Keywords:** Effective communication, Aged, Patient outcomes

## Abstract

**Background:**

Effective communication is a cornerstone of quality healthcare. Communication helps providers bond with patients, forming therapeutic relationships that benefit patient-centred outcomes. The information exchanged between the provider and patient can help in medical decision-making, such as better self-management. This rapid review investigated the effects of quality and effective communication on patient-centred outcomes among older patients.

**Methods:**

Google Scholar, PubMed, Scopus, CINAHL, and PsycINFO were searched using keywords like “effective communication,“ “elderly,“ and “well-being.“ Studies published between 2000 and 2023 describing or investigating communication strategies between older patients (65 years and above) and providers in various healthcare settings were considered for selection. The quality of selected studies was assessed using the GRADE Tool.

**Results:**

The search strategy yielded seven studies. Five studies were qualitative (two phenomenological study, one ethnography, and two grounded theory studies), one was a cross-sectional observational study, and one was an experimental study. The studies investigated the effects of verbal and nonverbal communication strategies between patients and providers on various patient-centred outcomes, such as patient satisfaction, quality of care, quality of life, and physical and mental health. All the studies reported that various verbal and non-verbal communication strategies positively impacted all patient-centred outcomes.

**Conclusion:**

Although the selected studies supported the positive impact of effective communication with older adults on patient-centred outcomes, they had various methodological setbacks that need to be bridged in the future. Future studies should utilize experimental approaches, generalizable samples, and specific effect size estimates.

**Supplementary Information:**

The online version contains supplementary material available at 10.1186/s12913-023-09869-8.

## Introduction

Excellent communication is critical for all health professionals [1, 2]. It affects the quality of healthcare output, impacts the patient’s health and satisfaction, and benefits both patients and providers [3]. Communication is a critical clinical competence because it establishes trust between providers and patients, creating a therapeutic relationship [4]. Physician-patient communication plays several functions, including making decisions, exchanging information, improving the physician-patient relationship, managing the patient’s doubts, addressing emotions, and enhancing self-management [5]. Features of effective or quality communication include involving patients in decisions, allowing patients to speak without interruptions, encouraging a patient to ask questions and answering the questions, using a language that the patient understands, paying attention to the patient and discussing the next steps [5]. This communication also includes listening, developing a good interpersonal relationship, and making patient-centred management plans.

The quality of patient-physician communication influences various patient-centred outcomes [6]. In this review, patient-centred outcomes refer to all the outcomes that contribute to the recovery or indicate the recovery of patients, as well as suggest positive experiences with the care process. For instance, effective communication is associated with enhanced patient satisfaction, regulating emotions, and increasing compliance, leading to improved health and better outcomes [7, 8]. According to [9], quality communication enhances patients’ trust in their providers, making patients more satisfied with the treatment. A trusting provider-patient relationship causes individuals to believe they receive better care [10]. For instance, [11] report that effective provider-patient communication improves social, somatic, and psychological health. During communication, the provider may enhance positive motivations and involve the individual in treatment decisions. Communication helps patients to acknowledge their illnesses, the associated risks, and the advantages of consistent treatment [5]. note that mutual communication between providers and patients stimulates or strengthens patients’ perception of control over their health, the knowledge to discern symptoms and self-care and identify changes in their condition. Effective communication leads to improved perceived quality of health care [12]. report that physician-patient communication influences the perceived quality of healthcare services. All these outcomes that suggest or contribute to patient’s positive experiences or imply a positive recovery journey, such as shorter hospital stays, are considered patient-centred outcomes.

This rapid review aims to review studies that have previously investigated the influence of quality communication on patient-centred outcomes among older adults, such as psychological well-being, quality of health care, emotional well-being, cognitive well-being, individualised care, health status, patient satisfaction, and quality of life. The specific objectives include (a) exploring the strategies used to ensure quality and effective communication with older patients in various healthcare settings, (b) exploring the patient-centred health outcomes reported by previous studies investigating quality communication between providers and older patients, and (c) to link quality communication strategies with older patients to patient-centred health outcomes among older patients.

The primary rationale for conducting this rapid review is that although many studies have examined the relationship between quality communication and various patient-centred outcomes, few studies have used older patients as their participants. It is a significant research gap because older adults have unique communication needs, which, if not considered, their communication with healthcare providers could be ineffective [13]. For example, older adults experience age-related changes in cognition, perception, and sensation, which can interfere with the communication process [14]. As a result, more research is needed to the specific quality communication strategies that could improve patient-centred outcomes among older adults. To my knowledge, no systematic review has focused on this topic. Therefore, this is the first rapid review to explore quality communication and its impact on patient-centred health outcomes among older patients in various healthcare settings.

This rapid review’s findings could inform practitioners of the quality communication strategies they can use to improve patient-reported outcomes. Besides, the rapid review evaluates the quality of studies investigating this matter and makes informed recommendations for future research to advance knowledge on this subject.

## Methods

This rapid review was conducted in conformity with the PRISMA (Preferred Reporting Items for Systematic Reviews and Meta-Analyses) guidelines [15]. The main difference between a systematic review and a rapid review is that the former strictly conforms to the PRISMA protocol, whereas the latter can miss a few elements of a typical systematic review. A rapid review was suitable because a single reviewer was involved in the study selection process, whereas at least two independent reviewers are recommended in typical systematic reviews [16].

### Eligibility criteria

Table 1 below summarises the inclusion and exclusion criteria used to guide study selection in this rapid review. Also, justification is provided for each inclusion/exclusion criteria. The inclusion/exclusion criteria were drafted based on the target population, the intervention, the outcomes, year of publication, article language, and geographical location. This approach corresponds with the PICO (P – population, I – intervention, C – comparison, and O – outcomes) framework [17].

**Table 1:** Inclusion and exclusion criteria

Inclusion	Exclusion	Justification
Studies using a sample of older adults/patients (defined as 65 years and above) under the care of healthcare professionals	Studies using a sample of older adults with communication impairment (e.g., aphasia)	The rapid review focused on the quality of communication in older adults without hearing or speaking impairment.
Studies focusing on effective communication interventions, both verbal and non-verbal	Studies focusing on communicative interventions for patients with communication impairment	This rapid review focused on older patients without any hearing or speaking impairment.
Studies focusing on patient-centred outcomes (variables promoting or indicating the patient recovery journey, or implying the patient’s subjective experiences of the care process), such as psychological well-being, quality of health care, emotional well-being, cognitive well-being, individualised care, health status, patient satisfaction, and quality of life.	No outcome reported	Focusing on a particular outcome like patient well-being would not have yielded any studies enough for review to address the review objectives due to the scarcity of research on this subject matter
Studies published between 2000 and 2023	Older studies	This review aimed to capture latest developments, advancements, and findings in the field. As a result, studies published within the past ten years were preferred. However, only a few articles were published within that timeframe, requiring the need for adjusting the timeframe to 2000–2023 to identify sufficient studies for review.
Studies published in the English language	N/A	The researcher is an English speaker, which means studies published in non-English languages could have resulted in translational errors and costs undermining the study’s credibility.
Primary studies using either qualitative designs, quantitative designs, or both	Secondary studies, like other literature reviews. Also, studies not reporting their methodologies at all.	The inclusion of secondary studies would have introduced bias into this rapid review.
Studies conducted in any country in the world	Studies conducted in sanctioned countries for violating international law norms and traditions, such as human rights violations	Considering the lack of research on this subject, focusing on a single country would not have yielded studies for review.

### Information sources

Four academic databases were searched: PubMed, Scopus, CINAHL, and PsycINFO. These databases were used as sources of information because they publish studies in healthcare sciences on a wide range of topics, including communication and the health outcomes of various interventions. Additionally, Google Scholar was searched to supplement the databases because it indexes academic journal articles in all disciplines, including healthcare. Combining Google Scholar with these databases has been recommended for an optimal search strategy [18].

### Search strategy

Various search terms related to the critical variables of this rapid review, namely quality communication, patient-centred health outcomes, and older patients, were combined using Boolean connectors (AND & OR). Regarding quality communication, some of the keywords that were used include “quality communication,“ “effective communication,“ “doctor-patient communication,“ and “patient-centred communication.“ The keywords that were used for patient-centred outcomes included “well-being,“ “patient satisfaction,“ “quality of care,“ “health status,“ and “quality of life.“ The search terms related to older patients included “nursing home residents,“ “older,“ and “elderly.“ Additionally, since most older patients are institutionalised, search terms like “nursing homes” and “assisted living facilities” were used in the search strategy. Table 2 below presents a sample search strategy executed on PubMed between September 2022 and July 2023. As shown in Table 2, Mesh terms were used alongside regular keywords. Truncations on the three keywords, namely elderly, nursing homes, and geriatric were used to allow more of their variations to be captured in the search. The use of Mesh terms was only performed on PubMed – Mesh terms are only supported on PubMed and MEDLINE. The rest of the sources of information were searched using the search terms without specifying whether they are Mesh terms or not.

**Table 2:** Search strategy

Search Terms	Date
a. (“quality communication” OR “effective communication” OR “doctor-patient communication” OR “patient-provider communication” OR “affective communication” OR “emotional support” OR “non-verbal communication“[Mesh] OR “facial expressions“[Mesh] OR “comfort touch” OR “therapeutic touch“[Mesh] OR “patient-centred communication”)	July 1, 2023
b. (“elderly*” OR “older” OR “geriatric*”)OR “nursing home residents” OR “aged”[Mesh])	July 3, 2023
c. (“nursing homes*“[Mesh] OR “long-term care facilities” OR “skilled nursing facilities“[Mesh] OR “assisted living facilities“[Mesh] OR “housing for the elderly”[Mesh])	July 4, 2023
d. (“psychological well-being“[Mesh] OR “quality of health care“[Mesh] OR “emotional well-being” OR “cognitive well-being” OR “individualised care” OR “health status“[Mesh] OR “patient satisfaction“[Mesh] OR “quality of life“[Mesh])	July 5, 2023
e. (a) AND (b) AND (c) AND (d)	July 6, 2023

### Study selection process

One reviewer (the author) was involved in screening the studies. The reviewer screened each record at least twice for confirmation purposes. Afterwards, an automation tool called ASReview which relies on machine learning to screen textual data was used as a second confirmation [19]. Research has shown that combining a machine learning tool and a single reviewer can significantly reduce the risk of missing relevant records [20]. This decision was reached based on previous research that has also demonstrated the good sensitivity of ASReview as a study selection tool in systematic reviews [19]. The software was trained on the eligibility criteria and the broader context of this study before it was used to screen the studies and confirm the reviewer’s decision. Therefore, if a record were retrieved, the author would screen for its eligibility the first time and confirm it the second time. For the third time confirmation, ASReview was employed. In case of disagreement between the author’s first and second attempts, a third attempt could be made to resolve it. In case of disagreement between the author’s first/second/third attempts and ASReview, a fourth attempt was made to resolve it.

### Data collection process

One reviewer (the author) extracted data from the qualifying records. The reviewer could collect data from a given study in the first round, record them, and confirm them in the second round. In case of disagreement between the first and second rounds, the author would extract data from the record for the third time to resolve it. The data points on which data extraction was based include the country where the study was conducted, the study’s research design (if reported), the population and setting of the study, the characteristics of the intervention (communication), and outcomes. Also, the author remained keen to identify ways the studies defined quality or effective communication in the context of older patient care. Regarding the characteristics of the intervention, some of the data sought included the type of communication (e.g., verbal or non-verbal) and the specific communicative strategies, such as touch and active listening.

Regarding outcomes, ‘patient-centred outcomes’ was used as an umbrella term for several variables that relate to the patient’s subjective well-being. Such variables include perceptions of quality of care, quality of life, symptom management, physical health, mental health, health literacy, patient satisfaction, individualised care, and overall well-being, including social processes, self-actualisation, self-esteem, life satisfaction, and psychosocial well-being. If studies reported on the acceptance and usability of communicative strategies, it was also included as a patient-centred outcome because the patient accepts a specific intervention and acknowledges its usability.

### Study quality assessment

The study quality assessment in this rapid review entailed the risk of bias and certainty assessments. Risk of bias assessment formed an essential aspect of certainty assessment. The risk of bias in qualitative studies was evaluated using the Critical Appraisal Skills Program (CASP) Qualitative Checklist [21]; the Cochrane Risk of Bias (RoB) tool was used for randomised studies [22]; and Risk of Bias in Non-Randomised Studies of Interventions (ROBINS-I) was used for cross-sectional observational studies [23]. The Grading for Recommendations, Assessment, Development, and Evaluation (GRADE) tool was used to assess the certainty of the evidence for all study designs [24]. The risk of bias in each study design and its corresponding assessment tool was calculated as a percentage of the total points possible. For example, the CASP Qualitative Checklist has ten items; each awarded one point. If a study scored seven out of 10 possible points, its risk of bias would be rated as 70%. The GRADE Tool has five domains, namely risk of bias, inconsistency, indirectness, imprecision, and publication bias. The first domain, risk of bias, was populated using the findings of risk of bias assessment using the stated tools. The overall quality of a study was based upon all five domains of the GRADE Tool.

### Synthesis methods

Both qualitative and quantitative studies were included in this review. The studies were highly heterogeneous in their research designs hence statistical methods like a meta-analysis synthesis were impossible [25]. Besides, the studies also had substantial heterogeneity in the study settings (some were conducted in primary care settings, but a majority were conducted in long-term care facilities/nursing homes) and outcomes. The studies measured different outcomes under the umbrella variable of patient-centred outcomes. As such, a narrative synthesis approach was considered the most suitable [26]. The narrative synthesis guidance by [27] was used. The first step based on the guidelines should be developing a theoretical model of how the interventions work, why, and for whom.

This rapid review’s explanation of how effective or quality communication leads to improved patient-centred outcomes in the introduction section formed the theoretical basis, that is, effective communication facilitates informational exchange between the patient and provider, leading to better decision-making, which positively influences patient outcomes The second step of a narrative synthesis entails organising findings from the included studies to describe patterns across the studies based on the direction of the effect size or effects [27]. The third step is to explore the relationship in the data by identifying the reasons for the direction of effects or effect size. This rapid review’s reasons were based on the theoretical notions outlined above in this paragraph. The final step is to provide insights into the generalizability of the findings to other populations, which, in the process, further research gaps can be outlined. The results are stated below.

## Results

### Study selection

**Fig. 1:**
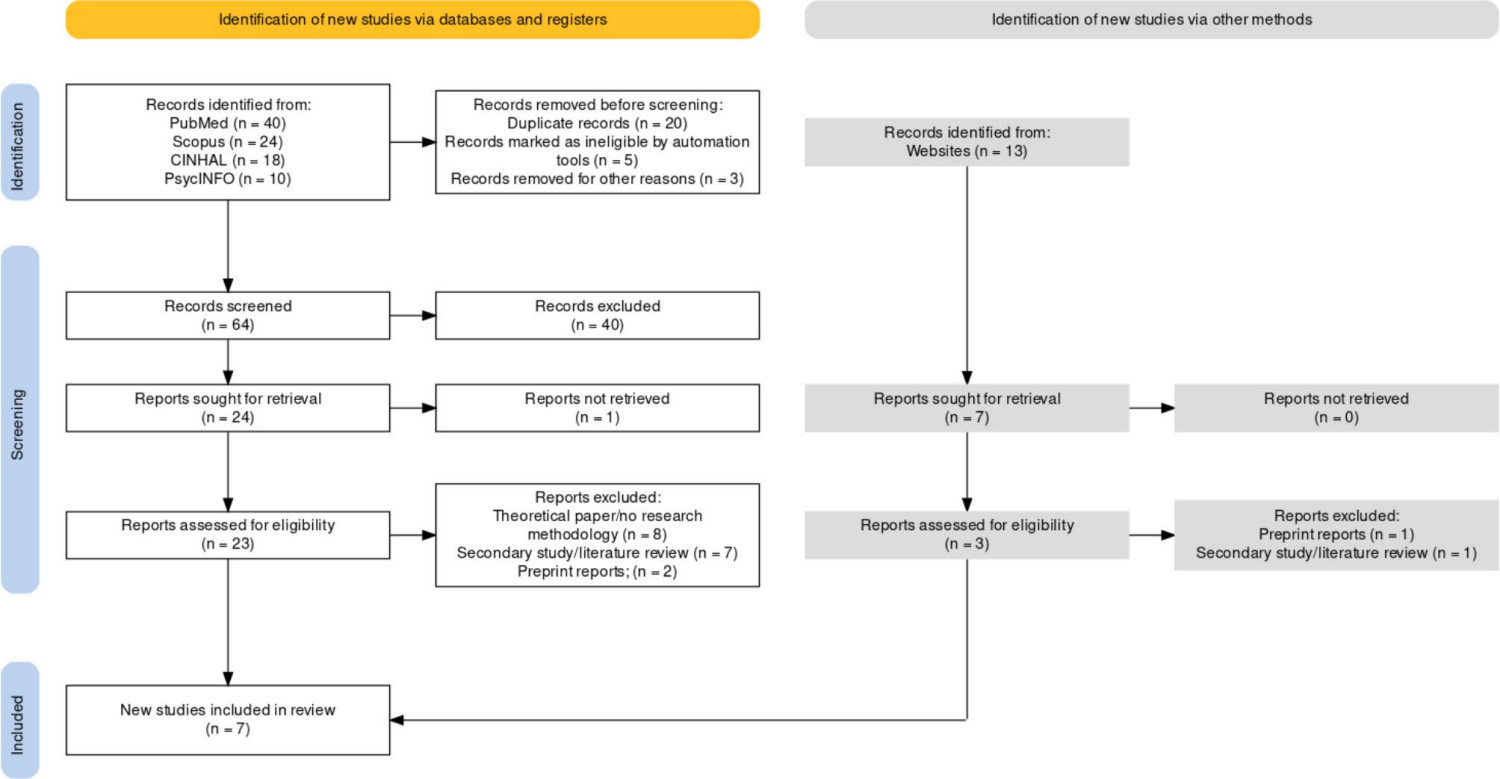
PRISMA Flowchart summarising the study selection process

After running the search strategy, 40 articles were identified from PubMed, 13 from Google Scholar (records identified from websites (Fig. 1)), 24 from Scopus, 18 from CINHAL, and 10 from PsycINFO based on the relevance of the titles. It was discovered that 26 were duplicated records between databases and Google Scholar, which reduced the number of identified records to 79. Further, the automation tool (ASReview) marked five records as ineligible based on their title considering the inclusion and exclusion criteria. These articles were excluded because the author confirmed in the fourth round that they were ineligible. After realising they did not focus on older adults, the author excluded three more records. Therefore, 71 records were screened using their abstracts with the help of ASReview (64 records from databases and 7 records from Google Scholar), whereby 44 were excluded (40 records from databases and 4 records from Google Scholar) for various reasons, such as being expert opinions and professional development based on field experiences (e.g., [28]) and did not have a methodology. The remaining 27 records (24 records from databases and 3 records from Google Scholar) were sought for retrieval, whereby one was excluded because its full text was inaccessible. The remaining 26 articles (23 records from databases and 3 records from Google Scholar) were assessed for eligibility with the help of ASReview, whereby eight records were excluded because they did not report their methodologies (e.g., [29]), another eight were secondary studies (e.g., [30]), and three were non-peer-reviewed preprints. Therefore, seven studies met the eligibility criteria for this rapid review.

### Study characteristics

Out of the seven studies, one was an experimental study [31], one was a cross-sectional observational study [32], and five were qualitative studies [33–37]. As shown in Table 3, most of the studies (n = 4) were conducted in the United States. The following countries produced one study each: Australia, Cameroon, the Netherlands, and Hungary. Although all the studies utilised a sample of older patients, the characteristics of the patients differed from one study to another. The studies ranged from primary care settings [36] and adult medical wards [37] to long-term care facilities like nursing homes. Apart from [36], the rest of the studies investigated various non-verbal communication strategies with older adults and their impact on various types of patient-centred outcomes, ranging from health-related outcomes (e.g., smoking cessation) to patient-reported outcomes, such as patient satisfaction, self-esteem, and life satisfaction. These outcomes are within the broader umbrella category of patient-centred m outcomes.

Further, the studies used different types of communicative strategies that can be used to enhance or promote patient-centred outcomes. In this rapid review, they were categorised into seven, namely (a) touching, (b) smiling, (c) gaze, head nod, and eyebrow movement, (d) active listening, (e) close physical distance, and (f) use of visual aids, and (g) telephone communication. Table 4 summarises the various ways in which each study described its interventions.

**Table 3:** Characteristics of included studies

Citation	Country	Research Design	Population and setting	Type of Communication/ Strategies	Outcomes
[31]	United States	Experimental	45 female older adults (65–101 years old) in two nursing homes in rural areas	Non-verbal (comfort touch)	Perceptions of self-esteem, well-being, health status, life satisfaction, social processes, and self-actualisation
[36]	Australia	Qualitative	44 COPD patients with a mean age of 65.2 years in primary care settings	Verbal (telephone)	Physical activity, smoking cessation, psychosocial well-being, symptom management, nutrition, and alcohol
[34]	The Netherlands and Hungary	Qualitative (developed a communication intervention for older adults using a formative approach, which started with a literature review, followed by focus groups and role-play exercises with older people to identify their communication needs, and finally developed storylines and sketches based on their findings before testing and retesting the intervention)	13 older adults with limited health literacy	Non-verbal (using visual aids like photos and video clips to support communication)	Acceptance of the intervention (whether appealing and comprehensible)
[32]	United States	Cross-sectional observational study with a follow-up assessment after four weeks	155 old patients (65 years and above) in nurse practitioners’ offices	Non-verbal (smile, touch, gaze, eyebrow movement, and nod or shake of the head)	Patient satisfaction, intention to adhere to nursing practitioners’ recommendations, changes in presenting problems, physical health, and mental health
[35]	United States	Qualitative	15 older patients in nursing homes	Non-verbal communication (listening and touching the shoulder)	Individualised care (authors hypothesised that it could lead to improve patient satisfaction, autonomy, and independence)
[33]	United States	Qualitative	17 older patients in nursing homes and assisted living facilities	Non-verbal communication (smiling, touching, laughing, eye gazing, shaking hands, head nodding, soft tone, and leaning forward)	Affective communication and patient’s well-being
[37]	Cameroon	Qualitative	Eight older patients in adult wards in two hospitals in Cameroon	Non-verbal communication (close physical distance, gentle touch, silence, and active listening)	Patient satisfaction and quality of care

**Table 4:** Description of interventions used in studies

Communication Strategy	Description
Touch	It can be a skin-to-skin touch for the sole purpose of comfort to foster positive feelings in elderly patients [31]; interpersonal touches, such as hugs, handshake, pat on the back, touching cheeks, or any other physical contact intended to communicate emotions or establish and maintain social bonds [32, 33, 37]; a pat on the shoulder to show the nurse cares [35].
Smiling	Smiles as a component of the relationship between the patient and the provider [32]; smiling when addressing the patient [33]; smiling as a communicative strategy to improve patient satisfaction with the services [37].
Gaze, head nod, and eyebrow movement	Gaze, head nods, and eyebrow movements as relationship components [32] or as nonverbal means of communication to address the patient [33], often combined with touch and smiling [32, 33].
Silence and active listening	Listening to patients as a way of showing them respect (combined with touching the shoulder) [35]; active listening as a channel of effective non-verbal communication [37].
Close physical distance or leaning forward	Close physical distance by sitting on patients’ beds and sitting close to patients [37]; leaning closer to the patient to look in their face [33].
Use of visual aids	Using photos and video clips to support communicative exchange between the patient and the provider is helpful, especially when patients have limited health literacy [34].
Telephone communication	Use of regular phone calls to promote behaviour change in patients using theoretical frameworks of behaviour change [36].

### Quality assessment findings

All seven studies were of high quality based on the GRADE Tool-based Assessment. However, [31] conducted an experimental study, but they did not provide any details indicating whether there was concealment in participant allocation and blinding of participants and outcome assessors. Therefore, it has a high likelihood of risk of bias. However, they scored excellently in the other domains of the GRADE Tool. All five qualitative studies and the cross-sectional observational study also scored excellently in the domains of the GRADE Tool, apart from the imprecision domain where they could not be scored because none of them reported effect sizes (Table 5).

**Table 5:** Quality assessment using the GRADE Tool

	Risk of Bias	Inconsistency	Indirectness	Imprecision	Publication Bias
[31]	High Risk	Low Inconsistency	Low Indirectness	Low Imprecision	Low Risk of Publication Bias
[36]	Low Risk	Low Inconsistency	Low Indirectness	N/A (Qualitative)	Low Risk of Publication Bias
[34]	Low Risk	Low Inconsistency	Low Indirectness	N/A (Qualitative)	Low Risk of Publication Bias
[32]	Low Risk	Low Inconsistency	Low Indirectness	N/A (No effect sizes reported)	Low Risk of Publication Bias
[35]	Low Risk	Low Inconsistency	Low Indirectness	N/A (Qualitative)	Low Risk of Publication Bias
[33]	Low Risk	Low Inconsistency	Low Indirectness	N/A (Qualitative)	Low Risk of Publication Bias
[37]	Low Risk	Low Inconsistency	Low Indirectness	N/A (Qualitative)	Low Risk of Publication Bias

### Results of individual studies

[31] was the only experimental study used in this rapid review investigating the effect of comfort touch on older patients’ perceptions of well-being, self-esteem, health status, social processes, life satisfaction, self-actualisation, and self-responsibility. The authors did not report the effect sizes but indicated that comforting touch had a statistically significant effect on each of the five variables. In summary, the authors suggested that comfort touch, characterised by a handshake or a pat on the shoulders, forearm, or hand, had a statistically significant positive impact on the various patient-centred outcomes reported in their study. For each variable, the authors used three groups, the first and second control groups and the third experimental group. After delivering the intervention, they investigated whether the scores of these variables changed between three-time points in each of the three groups. The first time point was the baseline data collected before intervention was initiated; the second was two weeks after baseline data; and the third was four weeks after baseline data. The authors found that in each of the five variables, the scores remained almost the same in the three-time points for the two control groups, but there were significant improvements in the experimental group (the one that received the intervention). For example, the self-esteem variable was measured using Rosenberg’s Self-Esteem Scale, with the highest attainable score of 40. In the first control group, the score remained 27.00, 27.27, and 27.13 for Time 1 (baseline), Time 2 (after two weeks), and Time 3 (after four weeks), respectively. The same trend was observed in the second control group. However, in the experimental group, the score improved from 29.17 at baseline to 36.00 at Time 2 and 37.47 at Time 3. These findings suggest that comfort touch was highly effective in improving self-esteem among older patients. The same significant improvements were evident for all the other variables (p.184).

While all the other studies focused on nonverbal communication cues, [36] focused on telephone communication. They aimed to investigate the effect of a tailored intervention on health behaviour change in older adults delivered through telephone communication. Therefore, the primary rationale for selecting this study for review is that it used a specific communicative strategy (telephone) to deliver the intervention, which is the primary purpose of effective communication in most healthcare settings. The older patients used as participants in this study lived with COPD. The nurses trained to administer the intervention made regular phone calls over 12 months. The intervention was delivered to 90 participants. Of these, 65 were invited for interviews at the end of 12 months. One of the most important outcomes relevant to this rapid review is that the participants reported “being listened to by a caring health professional.“ It means that regular telephone communication improved the patient’s perceptions of the quality of care. Other critical patient-centred outcomes that improved due to this intervention include many participants quitting smoking and increased awareness of COPD effects.

[34] also conducted a qualitative study but needed to specify the specific research design, which was generally non-experimental. The authors used formative evaluation and a participatory approach to develop a communicative intervention for older adults with limited health literacy. In other words, apart from literature reviews, the authors involved the target population in developing a curated story to improve their health literacy. They developed photo and video-based stories by incorporating narrative and social learning theories. The most important finding of this study was that the authors found the developed communicative strategy appealing and understandable. Such observations imply that the participants’ health literacy also likely improved even though the authors did not evaluate it.

Further, using a sample of 155 older patients, [32] investigated the relationship between the communication characteristics between nursing practitioners and the older patients and patients’ proximal outcomes, namely patient satisfaction and intention to adhere to the NPs’ recommendations, and patients’ long-term outcomes (presenting problems and physical and mental health). The proximal outcomes (satisfaction and intention to adhere) were measured after visits, whereas the long-term outcomes (presenting problems, mental health, and physical health) were measured at four weeks. The communication and relationship components observed include various non-verbal communication strategies: smile, gaze, touch, eyebrow movement, head nod, and handshakes. The authors recorded videos during patient-provider interactions. These communicative strategies were measured using the Roter Interaction Analysis System (independent variable).

In contrast, the other outcomes (dependent variables) outlined above were each measured separately with a validated tool or single-item instruments [32]. For example, presenting problems were measured with a single-item instrument, whereas the physical and mental health changes at four weeks were measured using the SF-12 Version 2 Health Survey. The authors found that verbal and nonverbal communication strategies focused on providing patients with biomedical and psychosocial information and positive talk characterised by receptivity and trust were associated with better patient outcomes, such as significant improvements in mental and physical health at four weeks. Although the study did not report effect sizes, the findings agree that effective and quality communication can improve patient-centred outcomes like patient satisfaction.

[35] conducted a qualitative study with focus groups (eight focus groups with a range of three to nine participants) of 15 older adults in a nursing home. The study used an ethnographic qualitative design. The nonverbal communication strategies observed in this study included active listening (including verbal responses) and touching. The authors found that the characteristics of the communication strategies that make communication quality and effective include mutual respect, equity, and addressing conflict. The patients perceived that their nursing aides gave them better-individualised care if their relationship and communication were characterised by mutual respect. Portraying mutual respect includes showing the patients that they are being listened to and heard, which can include calling them by their names and showing signs of active listening. Some residents (older patients) complained that some nursing aides had favouritism, whereby they liked some patients and not others. When such a perception emerges, the patients could perceive the treatment as unjust, compromising individualised care quality. Also, nursing aides must equip themselves with communicative strategies to address conflict rather than avoid it. For example, knowing about the patient’s history can help nursing aides understand their behaviour in the facility, improving prospects of providing better personalised or individualised care.

[33] also conducted a qualitative study utilising a sample of 17 older adults in nursing homes and assisted living facilities in the United States. They aimed to identify the types and examples of nurse-aide-initiated communication with long-term care residents during mealtime assistance in the context of the residents’ responses. Using a naturalistic approach, the researchers observed communicative interactions between the nurse aides and the residents during mealtime assistance. Videos were recorded and transcribed and analysed using the grounded theory approach. They found that apart from emotional support, nonverbal communication strategies were used by nurse aides to address the residents, initiate and maintain personal conversations, and check-in. Although the authors did not provide statistical proof that these communication strategies improved well-being, their findings can inform future studies.

Finally, [37] conducted a qualitative, grounded theory study to develop a model for effective non-verbal communication between nurses and older patients. The authors conducted overt observations of patient-nurse interactions using a sample of eight older patients. They found that the nature of nonverbal communication to be employed depends on the context or environment, and certain external factors influence it. The factors influencing nonverbal communication include the nurses’ intrinsic factors, positive views of older adults, awareness of nonverbal communication, and possession of nonverbal communication skills. Patient factors that can also influence the effectiveness of nonverbal communication include positive moods, financial situations, and non-critical medical conditions. The model developed also emphasised that non-verbal communication, if carried out correctly considering context and environment, can lead to positive outcomes, such as increased adherence to providers’ recommendations, improved quality of care, and shorter hospital stays.

### Results of syntheses

Four themes emerged from the narrative synthesis: nonverbal communication, verbal communication, communication strategies, and patient-centred outcomes. Table 6 summarises the subthemes that emerged under each theme. They are discussed below.

#### Nonverbal communication

Nonverbal communication was a critical theme that emerged in several studies. Five out of the seven studies investigated the effectiveness of touch on various patient-centred outcomes [31]. found that nonverbal communication strategies such as comfort touch, characterised by a handshake or a pat on the shoulders, forearm, or hand, had a statistically significant positive impact on patient-centred outcomes, such as well-being, self-esteem, health status, social processes, life satisfaction, self-actualisation, and self-responsibility [31]. implemented comfort touch exclusively without combining it with other nonverbal communication strategies. It means that comfort touch on its own can be effective in improving various patient-centred outcomes. As such, it can be hypothesised that if comfort touch is combined with other nonverbal communication strategies, such as active listening, eye gazing, smiling, maintaining a close distance, eyebrow movement, and nodding/shaking of the head can lead to even better results regarding patient-centred outcomes [32, 33, 35, 37]. [35] identified active listening and touching as important nonverbal communication strategies that make communication quality and effective [33]. found that nurse-aide-initiated communication during mealtime assistance using nonverbal communication strategies, such as emotional support, smiling, laughing, touching, eye gazing, shaking hands, head nodding, leaning forward, and a soft tone were crucial in addressing the residents, initiating (and maintaining) personal conversations, and checking in. Finally, [37] developed a model that emphasised the importance of effective nonverbal communication in forming effective therapeutic relationships, promoting patient satisfaction, and improving the quality of care. An exhaustive list of the nonverbal communication approaches is shown in Table 6.

In general, most studies, especially the qualitative ones, supported the utilisation of multiple non-verbal communication strategies in a single communicative episode. The studies also implied that it is the responsibility of healthcare providers to initiate and maintain effective nonverbal communication cues, such as those detailed in Table 6. Additionally, it is important to note that it is only one study [31] that investigated the effectiveness of comfort touch on patient-centred outcomes. Therefore, the notion implied in qualitative studies that combining various nonverbal strategies could lead to a better improvement in patient-centred outcomes is subject to further empirical investigation. It was noted that there is a lack of empirical studies investigating how the combination of various non-verbal communication techniques or strategies can influence patient-centred outcomes, such as patient satisfaction and perceptions of quality of care.

#### Verbal communication

Four out of the seven studies implied that verbal communication improved patient-centred outcomes [32, 34–36]. Effective and quality verbal communication was found to impact patient satisfaction positively [32], increased awareness of COPD effects [36], improved health literacy [34], presented problems [32], and mental and physical health [32]. It is worth noting that [32] used a cross-sectional survey approach and used regression analyses to investigate the relationship between communication and various patient-centred outcomes, such as patient satisfaction and mental and physical health. Also, it is important noting that the authors combined both verbal (e.g., more positive talk, greater trust, and receptivity) and non-verbal (e.g., smile, gazing, eyebrow movements, and interpersonal touches) in their study. Therefore, it can be a bit challenging to directly conclude that effective verbal communication alone without non-verbal communication is effective on its own in improving patient-centred outcomes. Similarly, [34] combined both narrative-based and picture-based communication strategies to give patients education about health literacy. Therefore, it can be challenging to know whether narratives comprising of verbal communication (and often non-verbal communication) can improve patient-centred outcomes on their own. The rest of the studies were qualitative [35, 36], which means that their findings generally reflected the subjective experiences or opinions of their participants. Therefore, it can be said that although all the four studies supported verbal communication can effectively improve patient-centred outcomes, there is a need for future research to experimentally test its effectiveness without being combined with non-verbal communication strategies.

Moreover, two of the four studies implied that some conditions must be met for verbal communication to be effective [32, 35]. some communication strategies, such as higher lifestyle discussion and rapport-building rates, were perceived as patronising and associated with poor outcomes [32]. Instead, the authors found that communication strategies like seeking and giving biomedical and psychosocial information were more effective in improving patient outcomes [32]. It implies that healthcare providers should be attentive and intentional of the topics they discuss with patients. Further, in their qualitative study, [35] found that effective verbal communication also requires mutual respect, equity, and addressing conflict. Indeed, it appears that certain communication strategies like lifestyle discussions can undermine the process of establishing trust, which is why they were associated with adverse patient outcomes. Also, unlike nonverbal communication, the studies that highlighted the effect of verbal communication on patient-centred outcomes did not provide rich descriptions of the specific verbal communication strategies that can be used in a face-to-face healthcare setting. The described strategies like using phone calls to regularly communicate with the patient without having to visit a healthcare facility and things to ensure when communicating with the older patient, such as mutual respect and avoiding too many discussions on lifestyle do not offer rich insights into the specific nature of the verbal communication strategies.

**Table 6:** Subthemes

Nonverbal communication	Verbal communication	Communication Strategies	Patient-centred Outcomes
• Comfort touch [31] • Active listening [35] • Touching [35] • Smiling [32] • Gaze [32] • Eyebrow movement [32] • Head nod [32] • Handshakes [32] • Nurse-aide-initiated communication [33] • Emotional support [33] • Effective nonverbal communication [37]	• Verbal communication [32, 34, 36] • Biomedical and psychosocial information [32] • Positive talk [32] • Lifestyle discussion [32] • Rapport building [32] • Mutual respect [35] • Equity [35] • Conflict resolution [35]	• Tailored intervention [36] • Telephone communication [36] • Participatory approach [34] • Curated story [34]	• Well-being [31] • Self-esteem [31] • Health status [31] • Social processes [31] • Life satisfaction [31] • Self-actualisation [31] • Self-responsibility [31] • Patient satisfaction [32, 36] • Increased awareness of COPD effects [36] • Improved health literacy [34] • Presenting problems [32] • Mental health [32] • Physical health [32] • Adherence to providers’ recommendations [37] • Improved quality of care [37] • Shorter hospital stays [37]

#### Communication strategies

In 3.5.2 above, it was shown that the sample of participants that [32] used in their study did not prefer discussions related with healthy lifestyles, which compromised patient-centred outcomes. Therefore, it was also important to determine the best approaches to formulate communication strategies that work. Two out of the seven studies implied how communication strategies can be formulated [34, 36] [36]. found that a tailored intervention delivered through telephone communication improved patient perceptions of the quality of care. In this regard, the authors first identified the needs of the patients to guide the development of the tailored intervention, from which they might have obtained insights into the patients’ communication preferences [34]. found a participatory approach to developing a curated story that improves health literacy appealing and understandable. The findings emphasised the need for participatory approaches when developing communication interventions for patients with varied health and social needs. Although the studies did not compare or contrast the effectiveness of participatory-based communication strategies and non-participatory-based communication strategies, their findings provide useful insights into the significance of involving patients when developing them. From their findings, it can be anticipated that a participatory approach is more likely to yield better patient-centred outcomes than non-participatory-based communication strategies.

#### Patient-centred outcomes

All studies reviewed highlighted patient-centred outcomes as the goal of effective communication in older patients. Patient-centred outcomes included well-being, self-esteem, health status, social processes, life satisfaction, self-actualisation, and self-responsibility (Butt, 2001), as well as patient satisfaction [32, 36], increased awareness of COPD effects [36], and improved health literacy [34]. Others included presenting problems, mental health, and physical health [32], as well as adherence to providers’ recommendations, improved quality of care, and shorter hospital stays [37]. All seven studies indicated that the various verbal and nonverbal communication approaches could improve these patient-centred outcomes. The consistency observed between the experimental study by [31], the qualitative studies, and other quantitative study designs implies the need to pay greater attention to verbal and non-verbal communication strategies used by healthcare professionals as they can directly influence numerous patient-centred outcomes. This consistency further implies that effective communication is the anchor of high-quality care, and its absence will always compromise patient-centred outcomes, such as satisfaction and health outcomes.

## Discussion and conclusion

### Discussion of findings

In agreement with various studies and reviews conducted in younger populations [1–3], all the seven studies selected in this rapid review supported that effective communication is a cornerstone of improved patient-centred outcomes. Like [5, 11, 12], the studies reviewed in this rapid review also supported the idea that effective communication with older adults involves the combination of verbal and nonverbal communication cues. However, this rapid review went a step ahead to identify the specific conditions that must be present for effective verbal and nonverbal communication to take place, such as perceptions of equity, mutual respect, and addressing conflict instead of avoiding it. The qualitative studies used in this rapid review also offered rich descriptions of how providers use nonverbal communication strategies.

However, the main shortcoming of the seven studies reviewed is that none aimed to define or describe what constitutes effective communication with older adults, apart from [37], who described a model of nonverbal communication with older adults. The study was qualitative and only formed a theoretical basis of how effective nonverbal communication with older adults could be shaped. The theory developed needs to be tested in an experimental setting so that its effect size in improving patient-centred outcomes, such as quality of care, quality of life, patient satisfaction, and emotional and cognitive well-being, can be documented unbiasedly and validly. Therefore, as much as the reviewed studies agreed with younger populations regarding the positive effect of effective and quality communication on patient-centred outcomes [9, 10], the methodological rigour of studies with older patients needs to be improved.

Although the individual studies reviewed in this rapid review had low risk of bias apart from [31], the screening was based on the judgment of the individual research designs. Otherwise, if the assessment had been done from the perspective of the focus of this rapid review, the risk of bias in studies could have been high in predicting the influence of effective communication on patient-centred outcomes. First, apart from [31], none of the studies used a random sample. The qualitative studies used purposively obtained samples, which means the risk of bias from an interventional perspective was high. However, the studies provided in-depth insights into the characteristics and features of verbal and non-verbal communication strategies that can be used to form and maintain provider-patient relationships.

### Recommendations for practice and future research

The main recommendation for practice is that nurses and providers serving older patients must be aware of their verbal and non-verbal communication strategies. Besides, they should engage in continuous professional development to enhance their verbal and non-verbal communication skills. Combining a wide range of nonverbal communication, such as touching the patient on the shoulder or arm or even handshaking can help create strong bonds and relationships, which are key in an effective therapeutic relationship. The qualitative studies reviewed showed that nurses and other providers combine a wide range of nonverbal communication in a single interaction instance, such as eye gazing, nodding, touching, and eyebrow movement. Although studies on verbal communication were rare in this rapid review, some lessons learned from the few studies included (e.g., [36]) is that using telephones to communicate with older patients regularly is potentially effective in improving patient-centred outcomes like better self-management. The information shared by the nurse should be tailored to serve the specific health needs of older patients. For example, for COPD patients, a nurse can make regular calls to old patients to educate them about the importance of quitting smoking and alcohol to improve their health condition and better self-management. However, as [32] indicated, the nurse should be cautious about how to present the information to the client and be able to detect patronising discussions quickly. For example, the sample of adults used by [32] found that many lifestyle and rapport-building discussions with the nurse were patronising in ways that may be detrimental to patient-centred outcomes. Some of the strategies providers can employ to ensure that communication is not perceived as patronising by older patients include ensuring mutual respect (e.g., active listening as a sign of mutual respect), creating perceptions of equity rather than favouritism when communicating with multiple patients at a time, and solving conflicts rather than avoiding them, which entails extra efforts, such as understanding the patient’s behaviour in the past and present. Overall, although studies have not provided specific estimates of the effect sizes of effective communication on patient-centred outcomes among older adults, there is a general trend and consensus in studies that effective communication, nonverbal and verbal, is the cornerstone of high-quality healthcare.

Further, future research needs to address various gaps identified in this study. The first gap is that although [37] tried to develop a model of nonverbal communication with older adults, their study had some drawbacks that limited the comprehensiveness of the model. First, the authors used a sample of only eight older adults in two medical wards in Cameroon. Besides the small sample, the study was conducted in medical wards, which means its findings may not be generalisable to long-term care settings like nursing homes. More older adults who encounter healthcare professionals are admitted in long-term care facilities, calling for developing a more robust communication strategy. Second, [37] only focused on nonverbal communication, thereby providing limited practical applicability of the model since verbal and nonverbal communication co-exists in a single interactional instance. Therefore, there is a need to develop a model that provides a complete picture into what effective communication is like with older adults.

After developing a valid, reliable, and generalisable model for effective communication with older adults in various healthcare settings, future research should also focus on investigating the impact of such a model on patient-centred outcomes, such as quality of care, quality of life, patient satisfaction, and physical and mental health. More particularly, the developed model can be used to derive communication interventions, which can be applied and tested in various healthcare settings with older adults. That way, research on this subject matter will mature as more and more studies test the effectiveness of such a communication model in various settings and countries. All that is known in the literature is that effective verbal and nonverbal communication can help promote patient-centred outcomes among older adults.

### Limitations

Although this rapid review was conducted rigorously by adhering to the PRISMA guidelines, the use of a single reviewer in the study selection process can undermine the quality of the review. When a single reviewer is involved, the probability of missing out relevant studies increases immensely. However, this limitation was mitigated in this review by using an automation tool in the study selection process. In was assumed that combining the automation tool with one independent reviewer could significantly reduce the probability of missing relevant studies.

Another possible limitation is that few studies have been conducted between 2000 and 2023 investigating the effect of effective communication on various patient-centred outcomes. Although the literature recognises the importance of effective communication, and there is a unanimous agreement between studies of various research designs that it is the cornerstone of quality of care, more studies need to be conducted examining how various communication strategies influence patient outcomes, both subjective and objective. For example, [31] investigated the effect of comfort touch. Other studies using empirical means (e.g., experiments) can also test the other strategies identified, such as eye gazing, head nodding, eyebrow movement, et cetera. In this way, a more specific and structured approach to communication in healthcare settings can be developed using the evidence base.

Moreover, I initially intended to review studies published within the past five years (2018–2023) but later learned there were insufficient studies meeting the eligibility criteria. Consequently, I adjusted the publication date to the past ten years (2013–2023). I also learned insufficient studies published within that period. Consequently, I chose the period of 2000–2023, which yielded seven studies. Thus, some of the studies included may not capture contemporary realities in healthcare settings, raising the need for more empirical studies on this topic.

### Conclusion

This rapid review selected seven studies whose narrative synthesis demonstrated that effective verbal and non-verbal communication could improve patient-centred outcomes. However, the studies were mostly qualitative, and hence they only provided rich descriptions of how nurses and older patients communicate in various clinical settings. It is only one study (Butts, 2001) that was experimental. Still, its risk of bias was high since patients were not concealed to allocation, and participants and outcome assessors were not blinded. Future research needs to focus on deriving a valid, reliable, and generalisable communication model with older adults using a larger and more representative sample size of older patients. Such a model should encompass both verbal and nonverbal communication. After developing a robust model, the next phase of future studies is to derive interventions based on the model and then, through experimental research, test their effectiveness. In that way, a standard approach to communicating effectively and in quality will be achieved, which is yet to be achieved in the current studies.

### Electronic supplementary material

Below is the link to the electronic supplementary material.

Supplementary Material 1

## Data Availability

All data generated or analysed during this study are included in this published article [and its supplementary information files].
